# Caffeic Acid Enhances the Anti-Leukemic Effect of Imatinib on Chronic Myeloid Leukemia Cells and Triggers Apoptosis in Cells Sensitive and Resistant to Imatinib

**DOI:** 10.3390/ijms22041644

**Published:** 2021-02-06

**Authors:** Giordana Feriotto, Federico Tagliati, Riccardo Giriolo, Fabio Casciano, Claudio Tabolacci, Simone Beninati, Mahmud Tareq Hassan Khan, Carlo Mischiati

**Affiliations:** 1 Department of Chemical, Pharmaceutical and Agricultural Sciences, University of Ferrara, 44121 Ferrara, Italy; giordana.feriotto@unife.it; 2 Department of Neuroscience and Rehabilitation, University of Ferrara, 44121 Ferrara, Italy; federico.tagliati@unife.it (F.T.); r.giriolo@gmail.com (R.G.); 3 Department of Translational Medicine and LTTA Centre, University of Ferrara, 44121 Ferrara, Italy; cscfba@unife.it; 4 Department of Oncology and Molecular Medicine, Istituto Superiore di Sanità, 00161 Rome, Italy; claudiotabolacci@tiscali.it; 5 Department of Biology, University of Rome “Tor Vergata”, 00133 Rome, Italy; beninati@bio.uniroma2.it; 6 Drug Discovery & Design, AuraDynamics AS, 9012 Tromsø, Norway; mthkhan2002@gmail.com

**Keywords:** caffeic acid, apoptosis, chronic myeloid leukemia, imatinib, resistance

## Abstract

Among the phenolic acids tested on the K562 cell line, a model of chronic myeloid leukemia (CML), caffeic acid (CA) was biologically active on sensitive and imatinib (IM)-resistant cells at micro-molar concentration, either in terms of reduction of cell proliferation or triggering of apoptosis. The CA treatment provoked mitochondrial membrane depolarization, genomic DNA fragmentation and phosphatidylserine exposure, hallmarks of apoptosis. Cell cycle analysis following the treatment with comparable cytotoxic concentrations of IM or CA showed marked differences in the distribution profiles. The reduction of cell proliferation by CA administration was associated with increased expression of two cell cycle repressor genes, *CDKN1A* and *CHES1*, while IM at a cytotoxic concentration increased the *CHES1* but not the *CDKN1A* expression. In addition, CA treatment affected the proliferation and triggered the apoptosis in IM-resistant cells. Taken together, these data suggested that CA induced the anti-proliferative effect and triggered apoptosis of CML cells by a different mechanism than IM. Finally, the combined administration of IM and CA at suboptimal concentrations evidenced a synergy of action in determining the anti-proliferative effect and triggering apoptosis. The ability of CA to potentiate the anti-leukemic effect of IM highlighted the nutraceutical potential of CA in CML.

## 1. Introduction

Chronic myeloid leukemia (CML) is a myeloproliferative neoplasm with an incidence of 1–2 cases per 100,000 adults [1]. The fusion of the Abelson gene (*ABL1*) on chromosome 9 with the breakpoint cluster region (*BCR*) on chromosome 22 generates the oncoprotein BCR-ABL, a constitutively active tyrosine kinase promoting cytokine-independent cell proliferation which determines an excessive accumulation of myeloid cells in hematopoietic tissues [2]. Imatinib mesylate (IM) was the first tyrosine-kinase inhibitor to receive the Food and Drug Administration (FDA) approval for the treatment of patients with CML [3]. Since the introduction of IM, the annual mortality in CML has decreased from 10–20% down to 1–2%. However, drug resistance is recurrent due to the genomic instability of *BCR-ABL*-positive hematopoietic stem cells [4]. Despite this, IM is considered the most cost-effective drug for treating newly diagnosed CML patients, especially after it lost patent exclusivity in 2016 [5]. Many molecules with synergic therapy effect with IM have been tested in clinical trials. These are drugs already used in monotherapy of leukemia but replaced by the advent of IM [6]. 

Foods of plant origin have attracted attention as a reservoir of bioactive substances with potential effects on health [7]. For example, apigenin, a common plant dietary flavonoid, revealed cytotoxic and apoptotic effects on imatinib-sensitive and resistant CML cells [8], suggesting that molecules of potential therapeutic interest for CML could be introduced daily with food. Other molecules widely present in fruit and vegetable foods, the phenolic acids, have therapeutic potential [9]. The most represented hydroxycinnamic acids, caffeic (CA), ferulic (FA), chicoric (DCTA), neochlorogenic (5-CQA) and chlorogenic (3-CQA) acids, showed therapeutic potential in a plethora of tumors at micro-molar concentration [10,11,12,13,14,15,16,17,18,19]. In particular, CA is present in several food sources (thyme, blueberries and apples) and represents the major phenolic compound in coffee drinks [20]. The administration of a pineapple vinegar enriched in CA led to a sharp reduction of tumor weight and volume in nude mice challenged with breast cancer cells [21], as well as the administration of CA enhanced the anti-tumor effect of paclitaxel in nude mice challenged with H1299 non-small-cell lung cancer cells [22], underlining the effectiveness of phenolic acids to delay cancer progression. 

In light of the promising anticancer effects of phenolic acids, their potential on chronic myeloid leukemia is unknown. Therefore, we studied the cytotoxic and apoptotic potential of phenolic acids in a cellular model system of chronic myeloid leukemia, the K562 cell line, and their possible synergism with IM. 

## 2. Results

### 2.1. Effects of Phenolic Acids on Chronic Myeloid Leukemia Cells’ Viability

**Figure 1:**
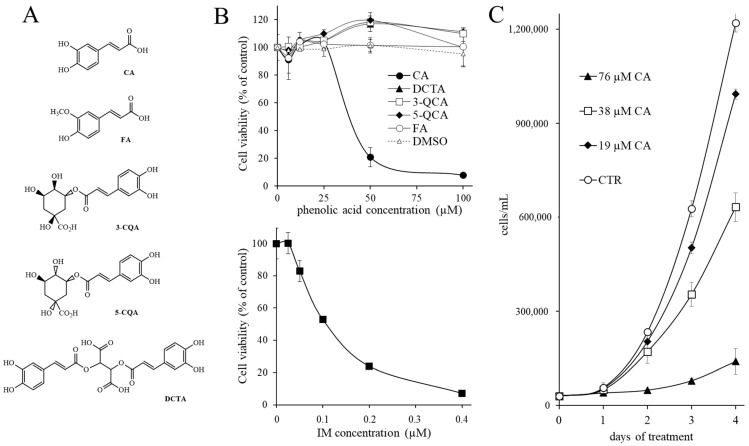
Effect of phenolic acids on the viability of the human K562 cells. (**A**) Structures of the molecules investigated. Caffeic acid (CA), ferulic acid (FA), chlorogenic acids (3-CQA), neochlorogenic acid (5-CQA) and chicoric acid (DCTA). (**B**) Cell viability evaluated by MTT assay three days after the drug administration, expressed as a percentage with respect to control cells left untreated. Upper panel: dose-dependent effects of phenolic acids. Lower panel: dose-dependent effects of imatinib (IM). (**C**) Time-dependent effects of CA on the number of viable cells assayed by Trypan blue dye exclusion. The results were expressed as arithmetic mean ± standard deviation.

The dose-dependent effects of CA, FA, DCTA, 5-CQA and 3-CQA (see structures in Figure 1A) on the viability of the human CML K562 cells were evaluated. The viability with respect to untreated cells was assessed by 3-(4,5-dimethylthiazol-2-yl)-2,5-diphenyltetrazolium bromide (MTT) staining 3 days after the drug administration. Cells were seeded in complete medium in the presence of phenolic acid (concentrations up to 100 µM) or equivalent amount of vehicle (dimethyl sulfoxide, DMSO). While CA affected the viability with IC_50_ = 38 μM, the other phenolic acids produced negligible effects, comparable with those of DMSO, and therefore were considered inactive in the range of concentrations tested (Figure 1B, upper side). The FA molecule, which has a structure very close to that of CA and is characterized by a single substitution of a hydroxyl with a methoxyl group in the aromatic ring, was inactive on CML cells and, therefore, it was used as a negative control drug in subsequent experiments. As expected, IM was strongly active with IC_50_ = 0.18 μM (Figure 1B, lower side). 

Figure 1C shows the effect of different CA concentrations on cell viability in the days following the addition to the culture medium. Cell viability was analyzed by the Trypan blue dye exclusion assay. While on the second day of treatment, the anti-proliferative effect of CA was appreciable, it was clearly evident on the third day for all the concentrations tested. 

### 2.2. Effect of CA Treatment on Cell Cycle Progression

**Figure 2:**
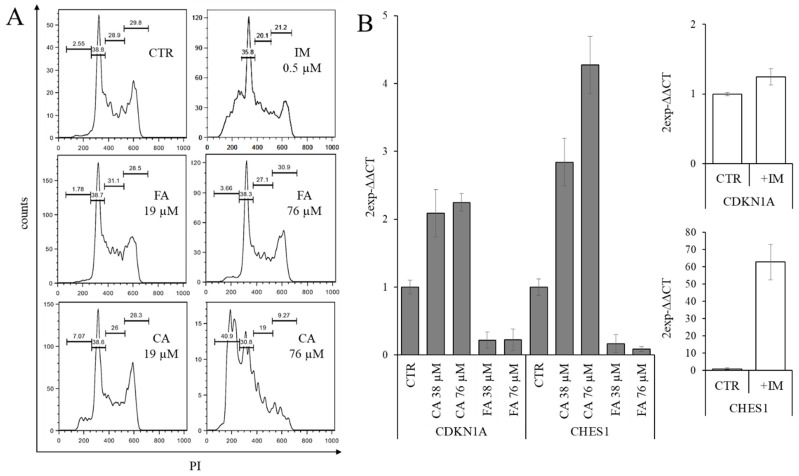
Effects of caffeic acid, ferulic acid and imatinib mesylate on cell cycle progression in K562 cells. (**A**) Effect on cell cycle distribution. The cells were cultured in complete medium for three days in the presence of the indicated compound. After staining with propidium iodide (PI), the cells were analyzed by flow cytometry. CA, caffeic acid; FA, ferulic acid; IM, imatinib mesylate. (**B**) RT-qPCR analysis of the expression of genes coding for cell cycle inhibitory proteins on the second day of treatment with the indicated concentration of CA or FA, or with 0.5 µM IM. CTR, untreated cells. The results were expressed as arithmetic mean ± standard deviation.

Therefore, the effects of similar concentrations of CA or FA on cell cycle distribution when administered to log-growing K562 cells for three days were analyzed (Figure 2). An aliquot of cells was processed by flow cytometry after RNase/PI staining (Figure 2A), and another one served in the analysis of the expression of cell cycle-related genes by reverse transcription-quantitative PCR (RT-qPCR) (Figure 2B). 

In Figure 2A, the treatment with suboptimal concentrations of CA (19 µM) resulted in a small augment of the percentage of sub-diploid cells with respect to FA-treated or untreated cells. This effect was better pronounced at a higher concentration (76 µM), markedly cytotoxic for CA but not for FA. At this CA concentration, cells in G0/G1 and S phases decreased, respectively, by 30% and 35% compared to untreated control cells, while those in the G2/M phase decreased dramatically by 70% but produced a massive increase in the sub-diploid peak. By contrast, the treatment with a comparable concentration of FA did not produce significant effects compared to untreated control cells, an expected result since FA did not affect proliferation at the concentrations tested. These results suggest that the effect of CA on cell proliferation could be triggered by increasing the expression of cycle inhibitory genes and facilitating cells to progress in the cell cycle towards cell death. 

Moreover, we studied the effects of administration of CA, FA or IM to K562 cells on the expression levels of negative regulator of cell cycle progression genes, the *CDKN1A* gene, which encodes for the potent cyclin-dependent kinase inhibitor p21 (WAF1/CIP1) [23], and the *CHES1* gene, which encodes for the forkhead transcription factor checkpoint suppressor 1 [24]. As showed in Figure 2B, which reports the RT-qPCR analysis data, the treatment with 0.5 µM IM strongly increased the expression of the *CHES1* mRNAs and produced a weak increase in *CDKN1A*, while the treatment with 38 and 76 µM CA increased the expression of both genes. By contrast, treatment with 38 and 76 µM FA did not increase the expression of these cycle-related genes, in accordance with the absence of effects on cell proliferation previously observed at these concentrations. These results suggested that the effect of CA on cell proliferation could be triggered by the increased expression of both of these inhibitory genes. 

### 2.3. Effect of CA Treatment on Apoptosis

**Figure 3:**
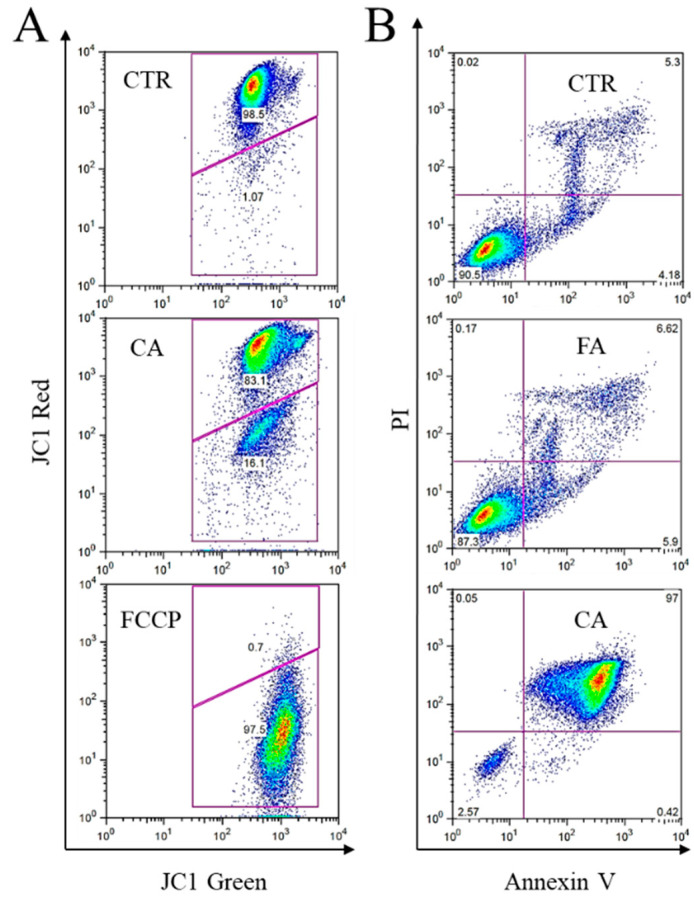
Effects of caffeic acid on triggering apoptosis in K562 cells. (**A**) A representative analysis of the mitochondrial membrane potential was performed by JC-1 staining on cells left untreated (CTR) or treated for two days in the presence of 38 µM CA. On the day of analysis, the control cells were split into two aliquots and the decoupling agent FCCP was added to one of them for 30 min to cause dissipation of the mitochondrial membrane potential. (**B**) A representative analysis of phosphatidylserine extroversion in cells on the third day of treatment with 76 µM CA or FA, cytotoxic and not cytotoxic, respectively. Cells were stained with Annexin V/PI. CA, caffeic acid; FA, ferulic acid; CTR, untreated cells.

An early aspect of apoptosis is mitochondrial destruction. It includes changes in the membrane potential due to the opening of the mitochondrial permeability transition pore. Therefore, we analyzed the effect of CA in inducing a loss of mitochondrial potential. The membrane-permeant JC-1 dye exhibits potential-dependent accumulation in mitochondria and is widely used to monitor mitochondrial depolarization, which is indicated by a decrease in the red/green fluorescence intensity ratio. To this purpose, K562 cells were treated in the presence of 38 µM CA or left untreated for two days and then stained with JC-1 and analyzed by flow cytometry (Figure 3A). In control cells left untreated, the red/green fluorescence intensity ratio was high. The following addition of the uncoupling agent carbonyl cyanide-4-(trifluoromethoxy)phenylhydrazone (FCCP) to the control cells produced a sharp decrease of red fluorescence and, consequently, of the red/green fluorescence intensity ratio. In cells subjected to treatment with CA, a percentage of them (about 16%) showed depolarization of the mitochondrial membrane, and consequently the opening of the mitochondrial permeability transition pore. 

In addition, we evaluated the apoptosis by phosphatidylserine extroversion after three days of CA treatment, by staining the cells with Annexin V/PI (Figure 3B) and flow cytometry analysis. As representatively shown in the figure, cells displayed phosphatidylserine extroversion and PI internalization, hallmarks of apoptosis. 

### 2.4. Synergistic Effect of CA and IM Treatment on Cell Proliferation and Apoptosis

**Figure 4:**
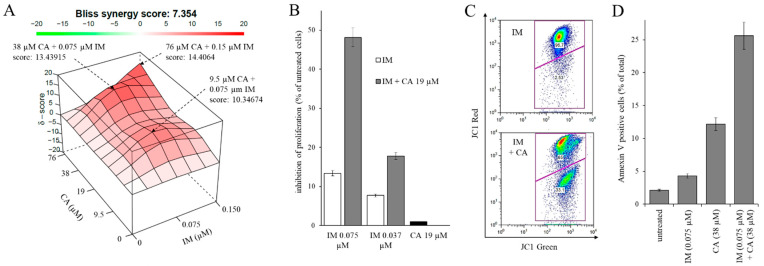
Synergistic effect of caffeic acid (CA) and imatinib mesylate (IM) on cell proliferation and on triggering apoptosis in K562 cells. (**A**) Calculation of the synergy scores. The indicated Bliss synergy score is the average of the values obtained for all the concentration combinations tested. The three-dimensional (3D) synergy maps highlight synergistic (in red) and antagonistic (in green) dose regions. The drug combinations with the most marked synergy scores have been indicated by the arrows. (**B**) Effect of combined treatment with suboptimal IM and CA concentrations on cell proliferation. Data are expressed as mean value plus standard deviation of three experiments performed in duplicate. (**C**) A representative analysis of the mitochondrial membrane potential was performed by JC-1 staining on cells treated for two days with 0.075 µM IM in the presence or absence of 38 µM CA. (**D**) Analysis of phosphatidylserine extroversion of cells treated with IM in the presence or absence of CA for three days. The results were expressed as arithmetic mean ± standard deviation. Cells were stained with Annexin V/PI.

The possible synergistic effect of CA (up to 38 µM) and IM (up to 0.15 µM) on K562 cell proliferation was evaluated through the calculation of Bliss synergy scores. The scores are reported in Figure 4A as a three-dimensional (3D) synergy map. The scores were always positive, with an average value of 7.354, indicating the synergic effect of the drugs at all the combinations tested. The highest scores were obtained by combining 9.5 µM CA + 0.075 µM IM (score: 10.34674), 38 µM CA + 0.075 µM IM (score: 13.43915) and 76 µM CA + 0.15 µM IM (score: 14.34064). In Figure 4B, we report the effect of suboptimal CA (19 µM) and IM (0.075 and 0.037 µM) concentrations on cell proliferation. At these concentrations, administration of IM had a small inhibitory effect, less than 13% compared to untreated control cells, while CA produced no inhibition at this concentration, as expected (see Figure 1B). In contrast, in the combined treatment of CA with lower or higher IM concentrations, a potent inhibitory effect on proliferation was observed, much greater than the sum of the cytotoxic effects obtained in the single treatment with each molecule. At the higher concentration of IM (0.075 µM), the combined treatment with CA increased the cytotoxic effect of the single molecule by more than three times, allowing it to reach inhibition values comparable to those obtained in a single treatment with concentrations 2.4 times higher of IM (0.18 µM, see Figure 1B). 

Furthermore, the possible synergistic effect of CA (38 µM) and IM (0.075 µM) on triggering apoptosis in K562 cells was evaluated. After two days of treatment, the polarization of the mitochondrial membrane was evaluated by staining with JC-1. A representative flow cytometry result is reported in Figure 4C. From the comparison of the data reported in Figure 3B and Figure 4B, although the administration of IM did not have an evident effect on the mitochondrial membrane potential, in the combined treatment, it showed a synergistic effect with CA, doubling the depolarizing effect of the latter (see Figure 3A). Furthermore, the extroversion of phosphatidylserine on the third day was assayed by Annexin V-fluorescein isothiocyanate (FITC) decoration (Figure 4D). Once again, the combined treatment with CA and IM produced an effect on apoptosis greater than the sum of the effects obtained with the individual molecules. 

Taken together, these data indicate a remarkable synergistic action of the two molecules in inhibiting cell proliferation and promoting the triggering of apoptosis in CML cells. 

### 2.5. Effect of CA on IM-Resistant K562 Cells

**Figure 5:**
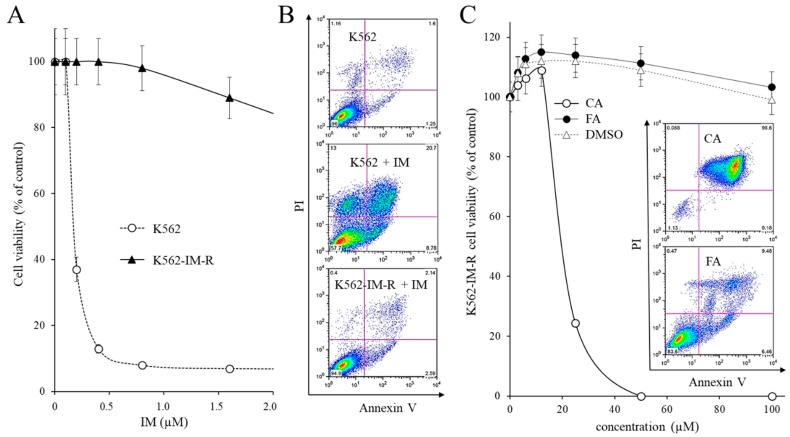
Effects of caffeic acid on viability and triggering apoptosis in IM-resistant K562. (**A**) Dose-dependent effects of IM on viability in sensitive (K562) and IM-resistant (K562-IM-R) cells three days after drug administration in the culture medium. (**B**) Representative flow-cytometer analysis of phosphatidylserine extroversion in sensitive K562 and K562-IM-R cells three days after the administration of 0.5 µM IM for three days. Cells were stained with Annexin V/PI. (**C**) Effect of CA on the viability of K562-IM-R cells three days after the addition in the culture medium. All treatments were performed in the presence of 0.5 µM IM in the culture medium. The results were expressed as arithmetic mean ± standard deviation. Insert: representative analysis of apoptosis carried out by staining with Annexin V/PI the cells treated in the presence of cytotoxic concentrations of CA or FA and 0.5 µM IM. CA, caffeic acid; FA, ferulic acid; IM, Imatinib mesylate.

The K562-IM-R cells, resistant to IM, were obtained in our laboratory, as described in the Materials and Methods Section. As shown in Figure 5A, K562-IM-R cells remained completely viable in the presence of 0.5 µM IM after three days of treatment, unlike the parental K562 cells which were sensitive to this IM concentration. The administration of 0.5 µM IM to K562-IM-R cells did not trigger apoptotic cell death, while, at the same concentration, had a powerful inducing effect of apoptosis on the sensitive parental line K562 (Figure 5B). 

The dose-dependent effect of CA or FA on the viability of K562-IM-R cells stably cultured in the presence of 0.5 µM IM was evaluated. The viability with respect to untreated cells was measured three days after the drug administration by MTT staining. As a control, equivalent amounts of DMSO (Figure 5C) were tested. CA affected cell viability with IC_50_ = 20 µM, about half of the value shown on K562 cells sensitive to IM, while FA produced negligible effects. The absence of IM in the culture medium did not change the cytotoxicity of CA (data not shown). These data indicated that CA has an even more marked anti-proliferative effect on IM-resistant cells with respect to the sensitive ones. Even in the K562-IM-R cells, unlike FA, CA has been shown to induce apoptosis, as demonstrated by the extroversion of phosphatidylserine (insert, Figure 5C). 

## 3. Discussion

Among the phenolic acids tested on the K562 model cell line of CML, CA was found to be active on sensitive and IM-resistant cells at micro-molar concentration, both in terms of reduction of cell proliferation and induction of apoptosis. It was observed that the reduction in proliferation caused by the treatment with CA was associated with increased expression of the two cell cycle repressor genes, *CDKN1A* and *CHES1*, which could represent the molecular switch underlying the anti-proliferative effect of the molecule. Since the IM at a cytotoxic concentration increased the *CHES1* but not the *CDKN1A* expression, it is reasonable to assume that CA and IM trigger different molecular mechanisms to determine the anti-proliferative effect, a hypothesis that could explain not only the synergism of action of the two molecules observed in Figure 3 and Figure 4 on sensitive cells, but also the anti-proliferative effect of CA in IM-resistant cells (see Figure 5). Further studies are needed to confirm this hypothesis. In addition, the different distributions observed in the cell cycle following the treatment with IM and CA concentrations comparable in terms of cytotoxicity suggested the activation of different mechanisms (see Figure 2A). Furthermore, the effect on cell proliferation was paralleled by the triggering of apoptosis. The intrinsic pathway is triggered in the early phase of apoptosis by increasing the permeability of the outer mitochondrial membrane through the opening of transition pores. As a result of this process, mitochondrial membrane potential decreases and cytochrome c moves from the intermembrane space into the cytoplasm to form the apoptosome [25]. In the later phase, apoptosome activates a cascade of proteolytic reactions leading to caspase-3 activation and responsible for membrane blebbing, DNA fragmentation and phosphatidylserine exposure, the last essential for apoptotic cells to be targeted by macrophages [26]. The data herein reported provided evidence that CA administration triggers the intrinsic pathway of apoptosis, since it provoked mitochondrial membrane depolarization, in agreement with data obtained by others in a non-blood cancer cell line [17], and also fragmentation of genomic DNA (increased pre-G0 sub-diploid pick in Figure 2A) and phosphatidylserine exposure. Taken together, the analysis of the effects produced by CA on the proliferation and triggering of apoptosis in sensitive and IM-resistant K562 cells suggested that this molecule has therapeutic potential in CML. Since the FA, a molecule very close in structure to the CA, did not produce equivalent effects on CML, the evidence suggested that the biological effects of CA were due to its characteristic structure and, therefore, this aspect should be duly taken into consideration in the design of future CA derivatives with improved activity on CML. Noteworthy, not only CA but also other natural products belonging to the flavonoid family exert anti-leukemic effects, e.g., quercetin, menadione and epigallocatechin-3-gallate [27,28], highlighting the potential of this family of molecules as a lead-compound in the development of future drugs for CML therapy. 

The data herein reported highlighted the nutraceutical potential of CA on CML, since the biological effect was observed at concentrations that are difficult to achieve with the diet. CA is the most widespread cinnamate in fruit and vegetables introduced with food, in particular as coffee drinks; also, the conjugated phenolic acids 5-CQA, 3-CQA and DCTA, being converted into CA in the gastrointestinal tract, represent an additional source of CA. Studies conducted on rats fed with a supplement of CA have shown that it is absorbed by the intestinal wall and reaches blood levels even much higher than those needed to trigger apoptosis in K562 cells, in concentrations ranging between 30 and 500 μM [9,29,30]. Considering that a complete meal in humans could introduce up to 500–800 mg of cinnamates (mainly CA) into the body [31], the amount of CA in the diet is largely insufficient to reach a concentration capable of exerting the biological effect we observed in vitro on K562 cells. From a speculative point of view, these considerations suggest that a diet rich in CA would not be therapeutically relevant in CML patients being treated with IM, since CA blood levels would be still too low to exert the synergistic effect with that drug. However, bioavailability studies carried out in rats have shown that effective levels of CA can be reached with a supplement of CA in the diet. Therefore, in this case, it is difficult to exclude a priori that CA may have a beneficial effect on CML in synergy with the IM in vivo. In this regard, it should be mentioned that there are examples in the literature in which the administration of CA in vivo has improved the antitumor effect of paclitaxel in nude mice challenged with H1299 lung cancer cells [22], which supports the potential ability of CA to enhance the effect of drugs used today in cancer therapy and to delay the progression of cancer in vivo also in the CML treatment. 

## 4. Materials and Methods

### 4.1. Materials

3-O-caffeoylquinic acid (3-CQA), 5-O-caffeoylquinic acid (5-CQA), dicaffeoyl-D-tartaric acid (DCTA), 3,4-dihydroxycinnamic acid (CA) and 4-hydroxy-3-methoxycinnamic acid (FA) were purchased from Sigma Aldrich (Milan, Italy). Stock solutions were prepared in dimethyl sulfoxide (DMSO) and stored in the dark at –20 °C until their use. IM was a kind gift of Novartis (Basel, Switzerland). Phosphate-buffered saline (PBS), cell medium and fetal bovine serum (FBS) were purchased from Euroclone (Euroclone SpA, Milan, Italy). 

### 4.2. Cell Culture

The K562 cell line, isolated from a patient with CML in terminal blast crisis [32], was cultured in Roswell Park Memorial Institute (RPMI)-1640 medium supplemented with 10% FBS, 100 U/mL penicillin and 100 μg/mL streptomycin at 37 °C in a humidified atmosphere of 5% CO_2_/air. 

### 4.3. MTT Assay

Cells in the log phase of growth were seeded at 25,000/mL into 96-well plates in fresh medium containing the examined compounds at various concentrations. After 72 h of growth, 25 μL of 0.5 mg/mL 3-(4,5-dimethylthiazol-2-yl)-2,5-diphenyl tetrazolium bromide (MTT) solution was added in the culture medium and the cells were incubated for 4 h at 37 °C. Then, 100 μL DMSO was added to each well to allow the formed formazan crystals to dissolve. Cells’ viability was assessed by the multi-well plate reader Infinite 200 PROTecan (TECAN, Mannedorf, Switzerland), measuring the absorbance at 570 nm. The proliferation in each sample was expressed as percent with respect to untreated control cells. Percentages were graphed as a function of molecule concentration and concentration required for 50% inhibition of the cellular proliferation, the half-maximal inhibitory concentration (IC_50_), was calculated. Three independent experiments were performed in triplicate. 

To calculate the degree of synergy of CA and IM, K562 cells were seeded in 96-well plates as described above and treated with different combinations of CA and/or IM. The effects on cell proliferation were assessed three days after by MTT assay. The mean value of three experiments conducted in triplicate were expressed as percent of viability and served as input file for the SynergyFinder online software (https://synergyfinder.fimm.fi, accessed on 20 January 2021) [33]. The degree of synergy was calculated as multiplicative effect of single drugs using the Bliss reference model. 

### 4.4. Mitochondrial Membrane Potential

Cells were washed twice and suspended in PBS containing 0.1 µM 5,5′,6,6′-tetrachloro-1,1′,3,3′-tetraethylbenzimidazolyl-carbocyanine iodide (JC-1) monomer (Cayman Chemicals, Ann Arbor, MI, USA) at a concentration of 5 × 10^5^ cells/mL and incubated at 37 °C for 15 min. Afterward, the cells were washed twice in PBS and fluorescence was acquired using a FACSCalibur flow cytometer (Becton-Dickinson, San Jose, CA, USA) equipped with a 488 nm air-cooled argon-ion laser, and then the data were analyzed using the FlowJo software (Tree Star Inc., San Carlos, CA, USA). In order to avoid spectral overlap, compensations using single-color controls were performed. JC-1 fluorescence was analyzed on the FL1 (530 nm band-pass filter) and FL2 (585 nm band-pass filter) channels for detection of the dye monomer and dimer form, respectively. 

### 4.5. RT-qPCR Analysis

RNA isolation was carried out by using guanidine isothiocyanate (TRIzol reagent, Invitrogen Corporation, Carlsbad, CA, USA). Cells were lysed in 1 mL of TRIzol reagent and 200 µL of chloroform was added. The mixture was vigorously shaken, incubated at room temperature for 10–15 min and centrifuged at 12,000× *g* for 15 min. The aqueous phase was collected, and the RNA was precipitated by isopropanol addition. The pellet obtained was washed in 75% ethanol and dissolved in 10 mM Tris-HCl pH 7.5, 1 mM EDTA. Purified RNA samples were processed with the DNA-free™ DNA Removal Kit (Invitrogen, Thermo Fisher Scientific, Milan, Italy) to remove contaminating DNA. RNAs (2 µg) were reverse transcribed (RT) with or without the ImProm-II™ Reverse Transcription System (Promega Italia, Milan, Italy) using oligo-dT primers in a standard 20 µL reaction. Then, 1 µL of this reaction mixture was used as a template in gene-specific amplification performed on a StepOnePlus Real-Time PCR System using the StepOne software v2.3 (Thermo Fisher Scientific). The primers used in the amplification of *CHES1*, *CDKN1A* and *ACTB* mRNAs have been previously described [34]. PCR amplifications were performed in a 50 µL volume containing 25 µL SYBR green PCR master mix (Thermo Fisher Scientific) containing the ROX internal passive reference dye, 0.5 µM of each primer, optimized MgCl_2_ concentration between 1.5 and 3 mM. All determinations were performed in triplicate wells. Samples in which the RNA was not retro-transcribed have given C_T_ values comparable to those obtained in the no template control well. Endpoint amplified products were subjected to melt curve analysis. Relative quantity of target transcript in the sample was calculate with respect to the reference *ACTB* mRNA using a comparative C_T_ (∆∆C_T_) method. The relative value was expressed as 2^−∆∆CT^. 

### 4.6. Cell Cycle Analysis

Cell cycle progression was investigated at the second day of treatment by flow cytometry after ethanol-fixation and propidium iodide (PI) staining. After washing in PBS, 5 × 10^5^ cells were fixed in 1 mL cold 70% ethanol at −20 °C for 20 min, washed twice in PBS and suspended in 0.2 mL PBS containing RNase A for 30 min at 37 °C. PI was subsequently added to each sample and samples were incubated in the dark for 30 min. The PI fluorescence of individual nuclei was measured and proportions of cells in the pre-G0, G0/G1, S and G2/M phase of the cell cycle were automatically calculated using Lysis II analysis software (Becton Dickinson Italia, Milan, Italy). 

### 4.7. Annexin V/Propidium Iodide Analysis of Apoptosis

To determine the phosphatidylserine externalization, a typical event occurring in apoptotic cells, cells cultured in the absence or presence of each compound for 3 days were dual-stained using the Annexin V Kit (MabTag, Friesoythe, Germany), according to the manufacturer’s protocol, and analyzed by flow cytometry. Briefly, 1 × 10^5^ K562 cells from each experimental point were collected by centrifugation and suspended in 90 µL of Annexin V binding buffer. Then, 5 µL of Annexin V conjugate and 5 µL of PI solution were added and cells were incubated for 20 min in the dark. After this time, 400 µL of Annexin V binding buffer were added and cells were collected by centrifugation at 400× *g* for 5 min and suspended in 100 µL Annexin V binding buffer. Samples were analyzed by flow cytometry using a FACSCalibur flow cytometer (Becton-Dickinson, San Jose, CA, USA) equipped with a 488 nm air-cooled argon-ion laser. Data were analyzed using the FlowJo software (Tree Star Inc., San Carlos, CA, USA). Early apoptotic cells with exposed phosphatidylserine but intact cell membranes bound only to Annexin V-FITC. Cells in late apoptotic stages were labeled with both Annexin V-FITC and PI, whereas necrotic cells were labeled with PI only. 

### 4.8. Selection of IM-Resistant K562 Cells

K562 cells initially sensitive to the drug were cultured in complete culture medium in the presence of 0.1 μM IM for 15 days. They were then diluted in fresh medium and grown in the presence of increasing IM concentration, doubling it every 15 days. After 4 months, resistant cells (K562-IM-R) were capable to proliferate in the presence of 2 μM IM. To maintain the resistance characteristics, the line was subsequently grown in constant presence of 0.5 µM IM. 

### 4.9. Statistical Analysis

Experiments were usually performed in triplicate for each data point and repeated at least three times. The results were expressed as arithmetic mean ± standard deviation. Statistical calculations were performed using a one-way analysis of variance (ANOVA) and the differences among groups were examined using the Bonferroni *t*-test. *p*-values < 0.05 were considered significant. 

## Abbreviations

chlorogenic acid, 3-O-caffeoyl quinic acid (3-CQA); neo-chlorogenic acid, 5-O-caffeoyl quinic acid (5-CQA); chicoric acid, dicaffeoyltartaric acid (DCTA); caffeic acid (CA); ferulic acid (FA).
